# Supplementary material for: Ultra-high spin emission from antiferromagnetic FeRh
Source: Nat Commun. 2024 Jun 11;15:4958. doi: 10.1038/s41467-024-48795-z (PMC11166917; doi:10.1038/s41467-024-48795-z)
Supplement: Supplementary file 1 — Supplementary Information [file 41467_2024_48795_MOESM1_ESM.pdf]

Supplementary Information: Ultra-high Spin Emission from Antiferromagnetic Metal FeRh

Dominik Hamara,[1] Mara Strungaru,[2] Jamie R. Massey,[3,4,5] Quentin Remy,[6] Xin Chen,[7] Guillermo Nava Antonio,[1] Obed Alves Santos,[1] Michel Hehn,[8] Richard F.L. Evans,[2] Roy W. Chantrell,[2] Stéphane Mangin,[9] Caterina Ducati,[7] Christopher H. Marrows,[3] Joseph Barker,[3,*] and Chiara Ciccarelli[1,†]

[1]Department of Physics, University of Cambridge, Cambridge, UK
[2]School of Physics, Engineering and Technology, University of York, York, YO10 5DD, United Kingdom
[3]School of Physics and Astronomy, University of Leeds, Leeds LS2 9JT, United Kingdom
[4]Laboratory for Mesoscopic Systems, Department of Materials, ETH Zurich, 8093 Zurich, Switzerland.
[5]Paul Scherrer Institute, 5232 Villigen PSI, Switzerland.
[6]Department of Physics, Freie Universität Berlin, 14195 Berlin, Germany
[7]Department of Materials Science and Metallurgy, University of Cambridge, Cambridge, UK
[8]Université de Lorraine, CNRS, IJL, F-54000 Nancy, France
[9]Institut Jean Lamour (UMR 7198), Université de Lorraine, Vandoeuvre-lès-Nancy, France

S1. TEMPERATURE DEPENDENCE OF THE CORRECTION FUNCTION

The temperature dependence of the THz emission in FeRh-Pt is the central part of our study. It is therefore very important to understand how the optical and electrical properties of our sample depend on temperature to correctly account for changes in the absorbed pump fluence, $A(T)$, and the efficiency of THz field outcoupling $C(T)$. To filter out the contribution of these two variables from our temperature analysis, we introduce a correction function, $G(T) = 1/(A(T)C(T))$, and define a renormalised THz emission amplitude $S_y^*(T) = G(T)S_y(T)$, where $S_y(T)$ is the measured amplitude.

To obtain $A(T)$ we calculate the total absorption of an optical laser light at 800 nm using the standard Transfer Matrix Method [1]. To do so, we require the refractive indices of each of the constituents of the sample stack for the corresponding optical wavelength. We take the refractive indices of MgO and Pt to have negligible dependence on temperature as $n_{\mathrm{MgO}}(800\text{ nm}) = 1.7$ [2] and $n_{\mathrm{Pt}}(800\text{ nm}) = 0.95 + 4.71i$ [3]. The temperature dependence of the refractive index of FeRh at the fixed angular frequency $\omega = 2\pi c/\lambda$ with $\lambda = 800$ nm, is given by [4]:

$$n^2_{FeRh}(T) = 1 - \frac{\omega_p(T)^2}{\omega^2 + 1/\tau(T)^2} + \frac{i\omega_p(T)^2/\omega\tau(T)}{\omega^2 + 1/\tau(T)^2} + \frac{i\sigma_{\mathrm{inter}}(T)}{\varepsilon_0\omega}. \quad (1)$$

$\tau(T) = \sigma_{\mathrm{DC}}(T)/(\varepsilon_0\omega_p^2(T))$ is the scattering time [4]. The temperature dependence of the DC conductivity of FeRh, $\sigma_{\mathrm{DC}}(T)$, is obtained from van der Pauw measurements. $\omega_p(T)$ is the plasma frequency, which we take as $\hbar\omega_p^{\mathrm{AFM}} = 1.8$ eV and $\hbar\omega_p^{\mathrm{FM}} = 5.5$ eV [4] in the AF and FM phases of FeRh, respectively. $\sigma_{\mathrm{inter}}(T)$ is the interband conductivity, which we take as $\sigma_{\mathrm{inter}}^{\mathrm{AFM}} = -i\omega\varepsilon_0 \times (0.74 + 0.58i)$ and $\sigma_{\mathrm{inter}}^{\mathrm{FM}} = -i\omega\varepsilon_0 \times (18.26 + 32.77i)$ [5].

To describe $\omega_p(T)$ and $\sigma_{\mathrm{inter}}(T)$ in the phase-transition region, we introduce a temperature dependent scaling parameter $\alpha(T)$, with $\alpha(T \ll T_{(\mathrm{AF-FM})}) = 1$ in the AFM phase and $\alpha(T \gg T_{(\mathrm{AF-FM})}) = 0$ in the FM phase. The temperature dependence of α is obtained from the renormalisation of the THz transmission data around the AFM/FM transition. The full temperature dependence of the plasma frequency and interband conductivity is then:

$$\omega_p(T) = \alpha(T)\omega_p^{\mathrm{AFM}} + (1 - \alpha(T))\omega_p^{\mathrm{FM}}, \quad (2)$$

and

$$\sigma_{\mathrm{inter}}(T) = \alpha(T)\sigma_{\mathrm{inter}}^{\mathrm{AFM}} + (1 - \alpha(T))\sigma_{\mathrm{inter}}^{\mathrm{FM}}. \quad (3)$$

The optical absorption temperature dependencies for the FeRh(30)/Pt(3.5) and uncapped FeRh(30) samples in the top-pumping geometry are plotted in Fig. S1(a) and (b) respectively. These $A(T)$ find that for the maximum pump

* j.barker@leeds.ac.uk
† cc538@cam.ac.uk

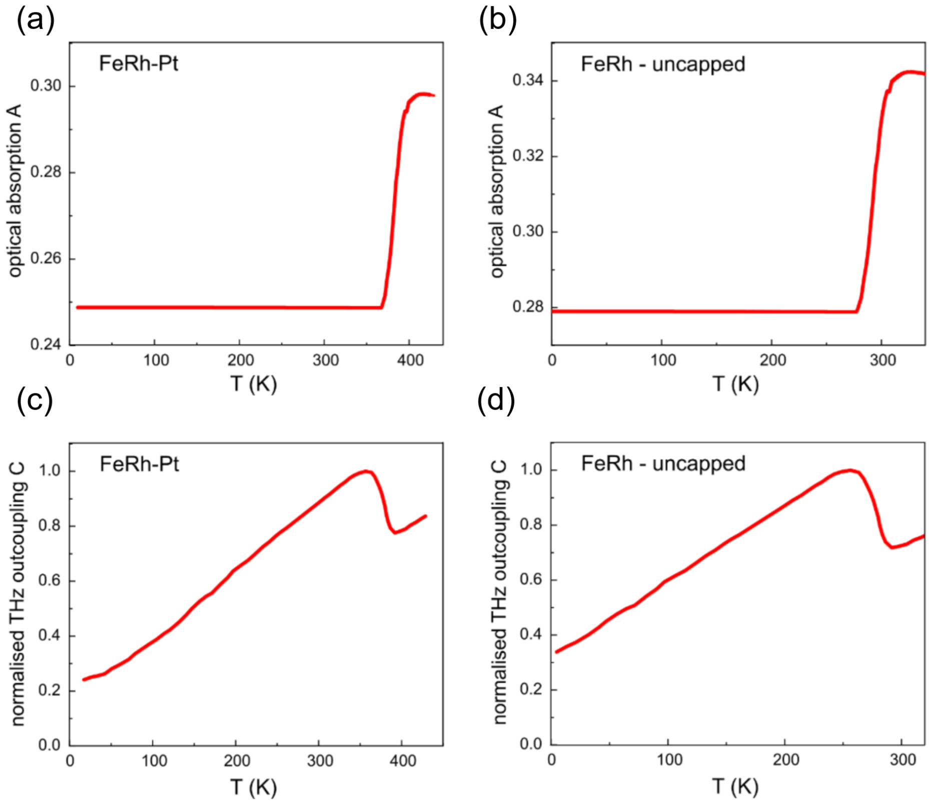

Fig. S 1 – Temperature dependence of the correction function parameters in the top pumping geometry: optical absorption fraction $A(T)$ **(a)** in FeRh(30)/Pt(3.5) and **(b)** uncapped FeRh (30); normalised THz outcoupling, $C(T)$ in **(c)** FeRh(30)/Pt(3.5) and **(d)** uncapped FeRh (30).

incident fluence of 9.52 mJ/cm^2, the absorbed fluences are in the ranges of 2.38-2.86 mJ/cm^2 and 2.67-3.28 mJ/cm^2 for FeRh(30)/Pt(3.5) and FeRh(30) respectively.

To find the temperature dependence of the THz field outcoupling at 1 THz, we use the expression given in Ref. 6:

$$C(T) \propto \frac{1}{1 + n_{\mathrm{MgO}} + Z_0 \int_0^d dz\ \sigma(T, z)}. \tag{4}$$

Here, $n_{\mathrm{MgO}} = 3.1$ [7] is the refractive index of the MgO substrate at 1 THz, considered to be temperature-independent. $Z_0 = 377\ \Omega$ is the vacuum impedance, d is the metal stack thickness, and $\sigma(T, z)$ is the conductivity distribution of the metal stack. At THz frequencies $\int_0^d dz\ \sigma(T, z) = d_{\mathrm{FeRh}}\sigma_{\mathrm{FeRh}}(T) + d_{\mathrm{Pt}}\sigma_{\mathrm{Pt}}(T)$, with $d_{\mathrm{FeRh}} = 30$ nm, and $d_{\mathrm{Pt}} = 3.5$ nm. The temperature dependence of the DC conductivities in FeRh [8] and Pt [9] are obtained from the literature. The normalised $C(T)$ for the FeRh(30)/Pt(3.5) and FeRh(30) samples are plotted in Fig. S1(c) and (d) respectively.

S2. THE NATURE OF THE RESIDUAL FERROMAGNETISM

A. Magnetic hysteresis loops of FeRh(30)/Pt(3.5) as a function of temperature

Fig. S2 shows hysteresis loops of FeRh(30)/Pt(3.5) measured using a SQUID magnetometer at temperatures between 400 K and 20 K. The MgO(001) background was removed using data sets obtained for a bare substrate. The paramagnetic contributions were subtracted. From these measurements we extract the temperature dependence of the saturation magnetisation M_{sat}, shown in Fig. 1(b) and 2(d) of the main text and of the coercive B_c and exchange bias B_{ex} fields in Fig. 2(d) of the main text.

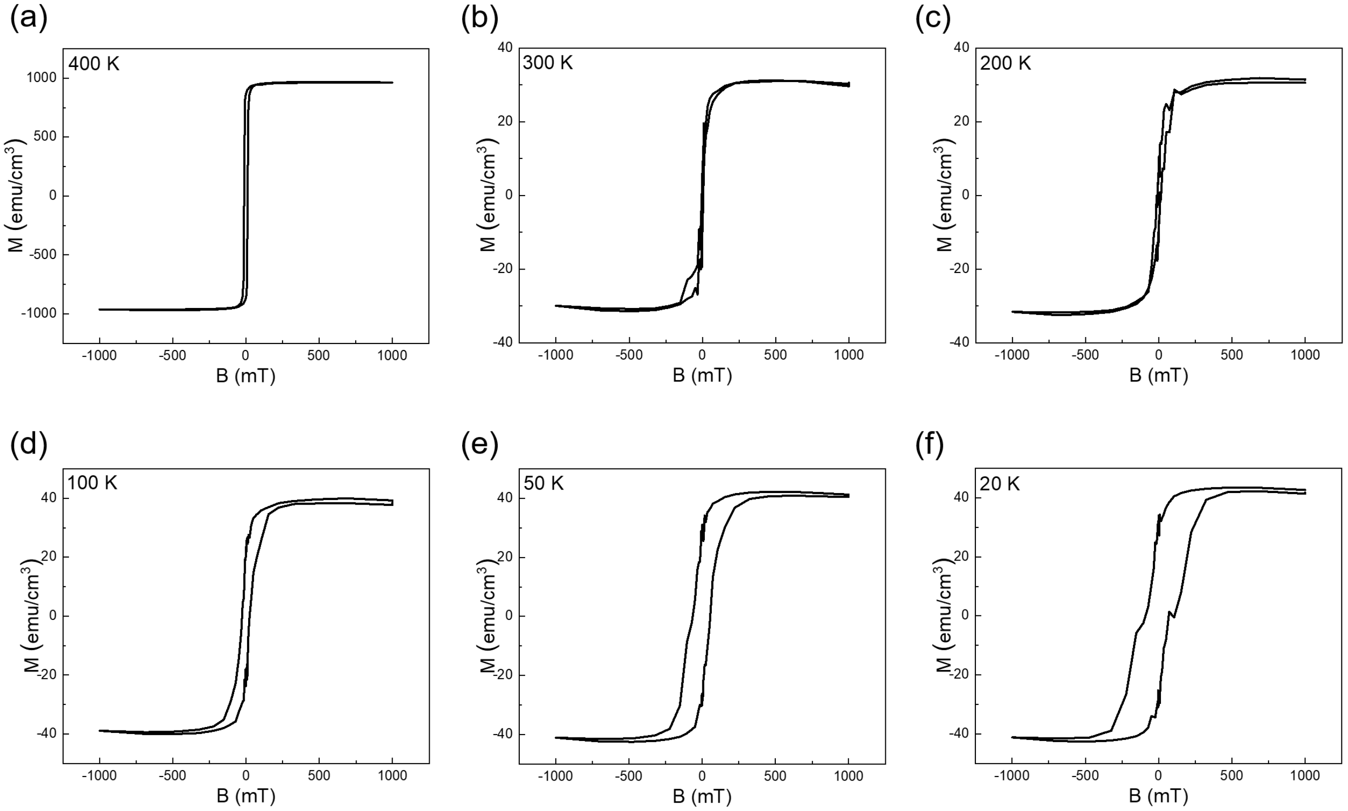

Fig. S 2 – Magnetic hysteresis loops of FeRh(30)/Pt(3.5) measured by SQUID at 400 K **(a)**, 300 K **(b)**, 200 K **(c)**, 100 K **(d)**, 50 K **(e)** and 20 K **(f)**, after subtracting the paramagnetic contribution and the contribution of the MgO(001) substrate. The saturation magnetisation and coercive field values in the main article were acquired from this data set.

B. Four-spin atomistic spin model

In this paragraph we want to show that the temperature dependence of the magnetisation measured by SQUID is reproduced by assuming a top layer of ferromagnetic FePd alloy. To model FeRh we used the atomistic spin dynamics software package VAMPIRE [10] that describes the dynamics of each spin, $\mathbf{S}_i$. The metamagnetic transition of FeRh is modelled in terms of the competition between the higher-order exchange interaction (four-spin) responsible for the antiferromagnetic ordering (AFM) at low temperatures and the bilinear exchange interaction, responsible for ferromagnetic ordering (FM) at elevated temperatures. The model has been developed by Barker and Chantrell [11] and further used in the literature [12, 13].

The spin Hamiltonian used in the atomistic simulations includes contributions from the bilinear exchange in form of nearest and next-nearest neighbors exchange and four-spin interaction and has the following form:

$$\begin{aligned}\mathcal{H} = &-\frac{1}{2}\sum_{i,j} J_{ij}\,(\mathbf{S}_i \cdot \mathbf{S}_j) - k_u \sum_i (\mathbf{S}_i \cdot \mathbf{e})^2 \\ &-\frac{1}{4}\sum_{i,j,k,l}\frac{1}{3}D_{ijkl}\,\left((\mathbf{S}_i \cdot \mathbf{S}_j)(\mathbf{S}_k \cdot \mathbf{S}_l) + (\mathbf{S}_i \cdot \mathbf{S}_k)(\mathbf{S}_j \cdot \mathbf{S}_l) + (\mathbf{S}_i \cdot \mathbf{S}_l)(\mathbf{S}_k \cdot \mathbf{S}_j)\right),\end{aligned} \tag{5}$$

where J_{ij} and D_{ijkl} represent the bilinear and four-spin exchange interaction between Fe atomic sites, respectively, k_u represents the uniaxial anisotropy constant, with $\mathbf{e}$ representing the easy axis direction. The factor $\frac{1}{2}$ accounts for the double summation $i \to j$ and $j \to i$ since numerically both interactions are included explicitly. Similarly, for the four-spin exchange term, the factor $\frac{1}{4}$ appears due to the explicit inclusion of all interactions for atoms i, j, k, l.

The parameters used in the simulations are presented in Supplementary Table 1, with the exchange parameters being summarised in Supplementary Table 2. The value of the uniaxial anisotropy was extracted from Ref. [12]. We

calculate the equilibrium magnetisation of the system by employing Monte-Carlo simulations, where we equilibrate the system at each temperature for 50,000 Monte-Carlo steps, and then average the properties for 50,000 more steps.

We modelled a system of 65 × 32 × 32 atomic sites, periodic in the yz directions, where at the FeRh/Pt interface we assume a FM monolayer of FePd. A reduced exchange (J_{FePd}) compared to the bulk FePd is used for the monolayer, an effect occuring due to the disordered state of the interface. The magnetic moment at the interface is also reduced to 0.45 μ_B, a reduction in the moment being also observed in literature [14]. From the SQUID data M_{sat} (20 K) is about 20 times smaller than M_{sat} (400 K), renormalising by the total volume of FeRh. If we consider that the FM interfaces extend over a combined thickness of 10 nm, we obtain that the magnetisation of the interfaces at low temperature is about 7 times smaller than in the ferromagnetic phase, hence the value of 0.45 μ_B. This is just a rough approximation because the ferromagnetic interface with the MgO substrate is of a different nature and its magnetic moment might be different. A coupling of 10% of the exchange value of FePd is used across the interface. The inclusion of the FM monolayer leads to a similar temperature dependence of magnetisation as observed experimentally, with a low Curie temperature of the monolayer (of 220K) - green curve in Fig. S3.

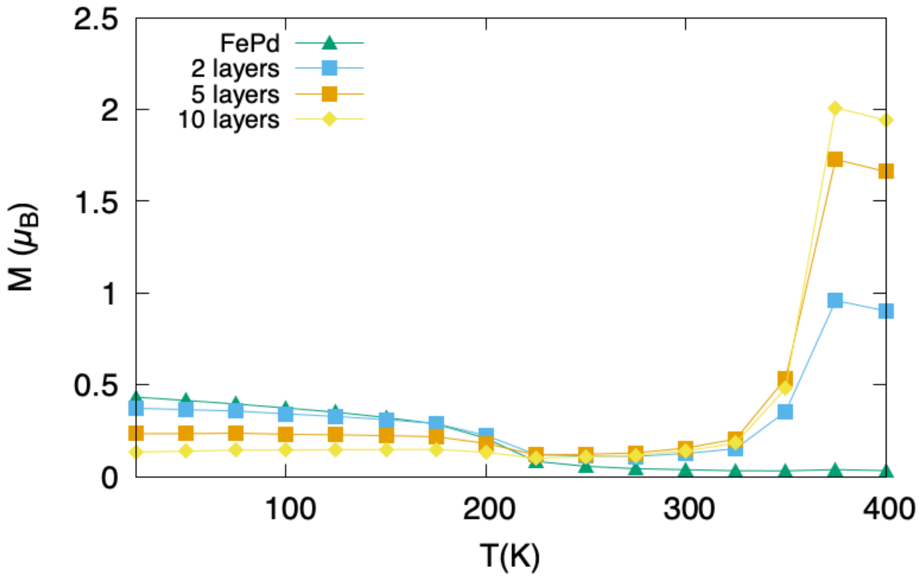

Fig. S 3 – Temperature dependence of magnetisation as extracted from atomistic spin dynamics simulations. The green curve shows the magnetisation of only the FePd monolayer. The blue, orange and yellow curves show the temperature dependence of the total magnetisation of 2, 5 and 10 monolayes of FeRh respectively plus a monolayer of FePd.

Supplementary Table 1 – Parameters used for the simulation of FeRh/Pt.

Quantity	Symbol	Value	Units
FeRh anisotropy energy	K_{FeRh}	1.404×10^{-23}	J
FePd anisotropy energy	K_{FePd}	2.63×10^{-22}	J
FeRh magnetic moment	μ_{FeRh}	3.15	μ_{B}
FePd magnetic moment	μ_{FePd}	0.45	μ_{B}

We next investigate the magnetisation behaviour arising from the various thickness of the considered interface. For an interface consisting of 2 layers (monolayer of FePd and monolayer of FeRh) blue curve in Fig. 3) we observe that the magnetisation in the low temperature regime has a similar amplitude that the one arising after the first order phase transition in FeRh. By distancing from the FePd interface, a decrease in the low temperature magnetisation is observed (as shown for 5 and 10 layers, orange and yellow curve).

The field dependence observed experimentally can appear as an effect of magnetic domains. For the simulation results presented above, the system size considered allows only for monodomain configurations at low temperature.

Supplementary Table 2 – Exchange constants for the four-spin exchange Hamiltonian (5).

symbol	types	number	distance (lattice constants)	value (meV)
J_2^{FeRh}	Fe-Fe	6	1	2.49[a]
J_3^{FeRh}	Fe-Fe	12	$\sqrt{2}$	17.16[a]
J_2^{FePd}	Fe-Fe	6	1	2.49
$J_2^{\mathrm{FeRh-FePd}}$	Fe-Fe	6	1	0.249
D_{ijkl}^{FeRh}	Fe-Fe-Fe-Fe	32	1	1.43[a]

[a] Ref. 11

However, for larger system sizes (which go beyond our computational capabilities), magnetic domains can be present at low temperature. Applying a magnetic field will saturate these domains in the direction of the field, hence the increased in the overall magnetisation along field direction can lead to an increse in the spin current with enhanced field as shown experimentally.

C. Scanning Transmission Electron Microscopy measurements

To confirm that residual ferromagnetism at the top interface originates from FePd alloying we carry Scanning Transmission Electron Microscopy (STEM) measurements on a 35 nm thick FeRh sample with a nominally uniform Pd doping of 2.8% across its thickness. The FeRh-Pd sample was coated with 30 nm sputtered amorphous carbon to protect the surface during the TEM lamella preparation. A cross-sectional lamella was cut from FeRh-Pd sample and thinned to approximately 100 nm to achieve electron transparency, following a standard protocol of FEI Helios Nanolab Dualbeam focused ion beam-scanning electron microscopy (FIB-SEM). The lamella was immediately transferred to a FEI Tecnai Osiris TEM equipped with an X-FEG and operated at 200 kV. STEM high-angle annular dark-field (STEM-HAADF) images were acquired using a beam current of around 1.05 nA, camera length of 115 mm, dwell time of 6 s and spatial sampling of 1 nm per pixel. STEM energy-dispersive X-ray spectroscopy (STEM-EDX) spectrum images were acquired with a Super-X system using a beam current of 1.05 nA, a dwell time of 800 ms and a spatial sampling of 3 nm per pixel. STEM-EDX data were denoised with principal component analysis (PCA) and quantified in Hyperspy, a Python-based toolkit for hyperspectral data processing [15].

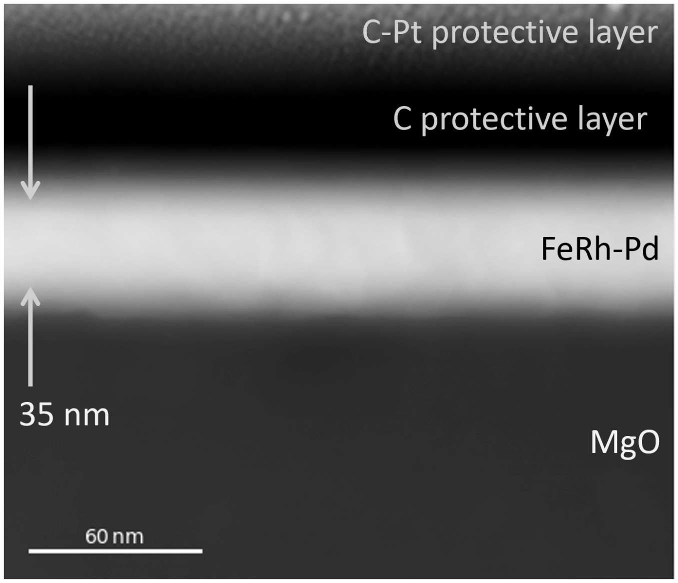

Fig. S 4 – STEM-HAADF image of FeRh layer on MgO substrate. The film is coated with protective C and C-Pt layerstively plus a monolayer of FePd.

The FeRh-Pd film appears as a bright layer in Fig. S4, where the signal intensity is proportional to lamella thickness and atomic number squared ($I \propto tZ^2$). The nominal thickness of the layer is 35 nm, but both interfaces

appear blurred, likely due to magnetic effects on the electron beam [16]. The elemental composition is quantified pixel-by-pixel using the Cliff-Lorimer method, with an accuracy estimated as 1% [17]. The HAADF navigator image and elemental ratio map for Pd/Fe are presented in Fig. S5 (a) and (b), respectively. The Pd/Fe ratio is constant across the film, but it increases by 4-6% at the top surface. Note that the larger apparent width of the FeRh-Pd film in (b) is due to the effect of the wider interaction volume for X-ray generation. For a thickness of approximately 100 nm of FeRh-Pd, the beam broadening is estimated as 6 nm (2 pixels). The variation of elemental ratios across the FeRh-Pd film are shown in Fig. S5 (c). Pd:Fe, Rh:Pd and Rh:Fe are normalized and plotted to highlight the compositional variation. The Pd content is constant across the film at ∼6.6 atomic percent in Fe. At the top surface there is an increase of about 5% in the Pd:Fe ratio while the Rh:Pd and Rh:Fe ratios decrease, which indicates a Rh-depleted and Pd-enriched top surface layer.

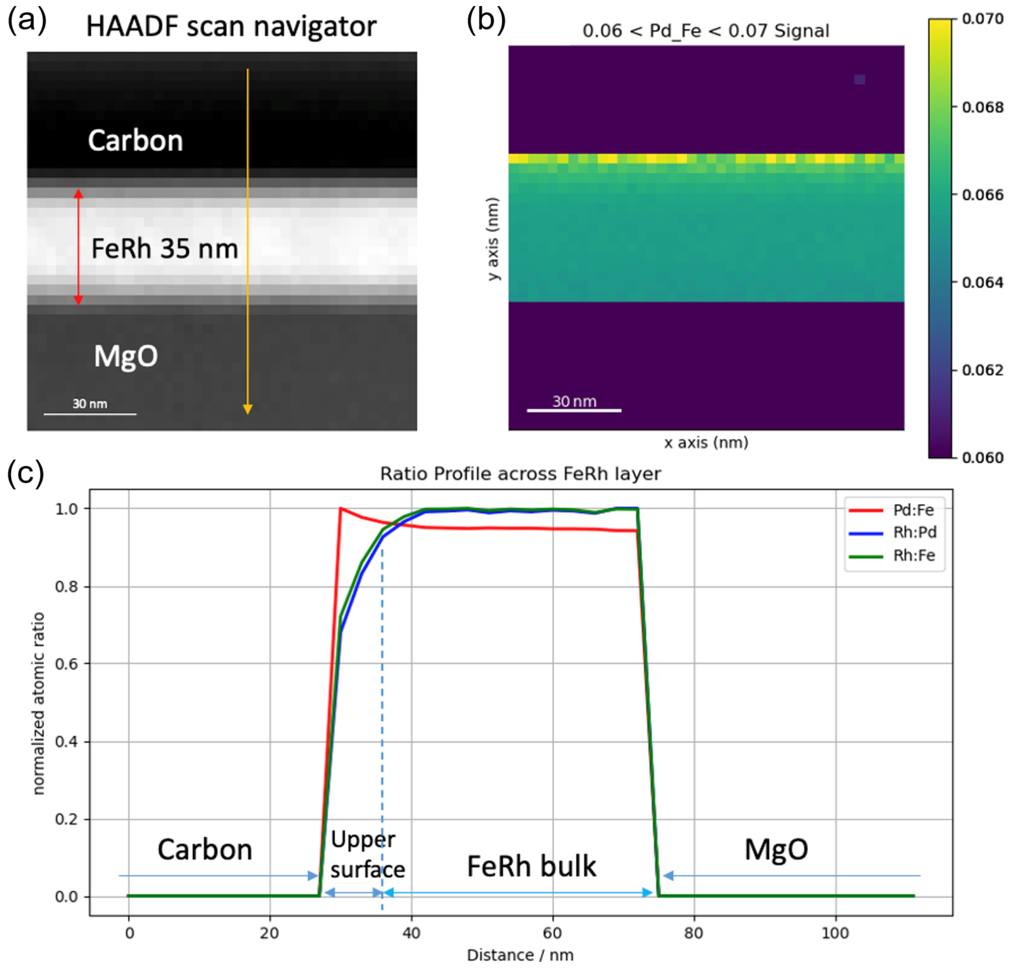

Fig. S 5 – Variation of elemental ratios across the FeRh-Pd film. (a) HAADF navigator for EDX scan, (b) Pd:Fe atomic ratio map, (c) Normalized ratio profile across FeRh layer for Pd:Fe, Rh:Pd and Rh:Fe.

S3. ADDITIONAL EXPERIMENTAL DATA

D. Raw THz emission data of FeRh(30)/Pt(3.5)

Fig. S6 presents the temperature and magnetic field dependence of the raw THz emission data, before renormalising by the temperature dependent optical absorption and temperature dependent THz outcoupling. The THz emission amplitude undergoes an abrupt decrease across the phase transition from ferromagnetic to antiferromagnetic. As temperature is further decreased, the THz emission amplitude starts increasing again up to 60% of the value in the ferromagnetic phase.

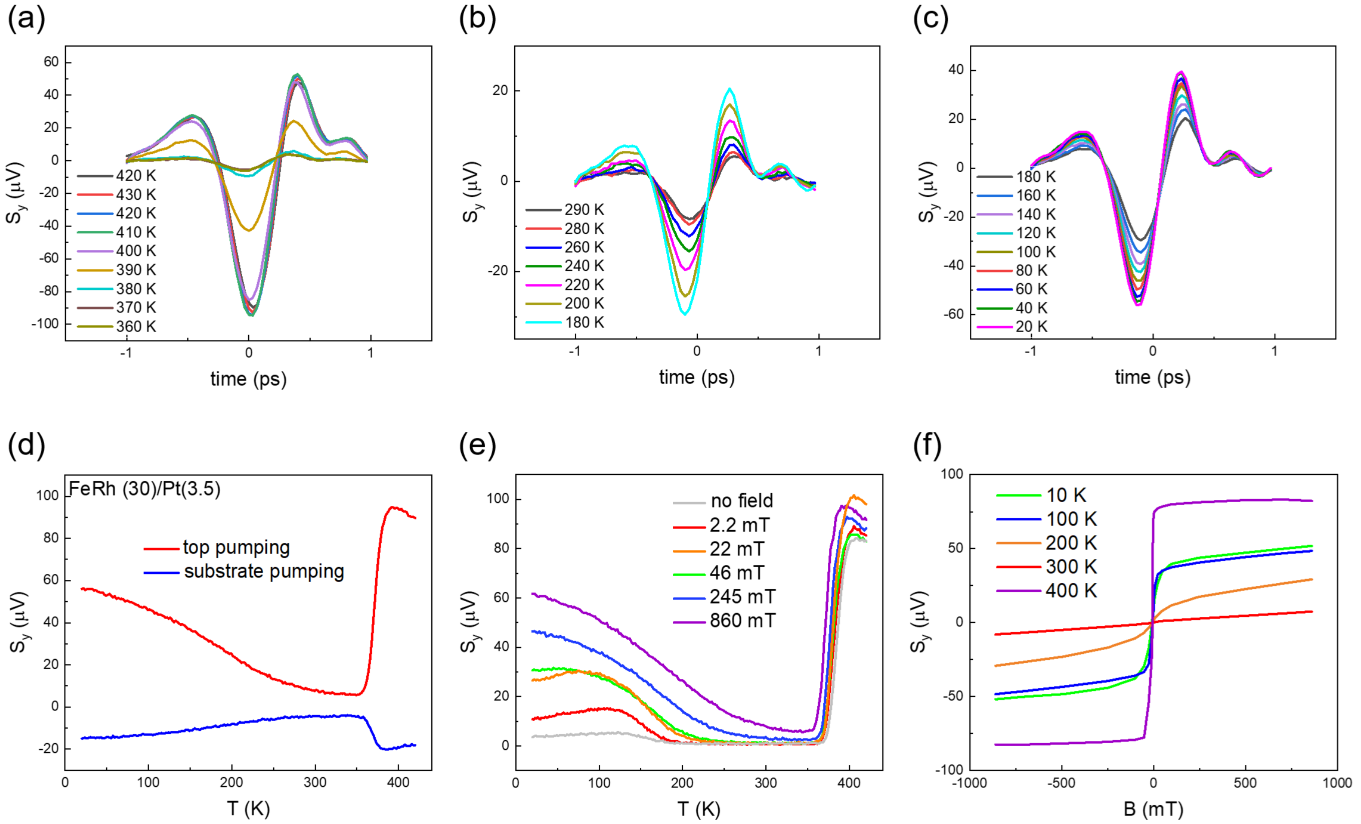

Fig. S 6 – THz emission data before renormalisation by the THz outcoupling and optical absorption. **(a)-(c)** THz transient time-traces measured at different temperatures in the range 420 K-360 K **(a)**, 290 K-180 K **(b)** and 180 K - 20 K **(c)**. **(d)** Temperature dependence of the THz emission amplitude measured at 860 mT for two pumping geometries, pumping the sample from the Pt side and measuring the THz emission behind the substrate (red), or pumping the sample from the substrate side and measuring the THz emission behind the Pt layer (blue). **(e)** Temperature dependence of the THz emission amplitude for different values of the in-plane magnetic field. **(f)** Dependence of the THz emission amplitude on the in-plane magnetic field at different temperatures. For these measurements a top pumping geometry was adopted, with the pump hitting the sample from the Pt side and the THz emission being measured behind the substrate.

E. Magnetic properties and raw THz signal of uncapped FeRh(30 nm)

The SQUID data in Fig. S7 (a) shows that in the uncapped FeRh(35) sample, residual ferromagnetism exists also in the antiferromagnetic phase and its saturation magnetisation reaches values comparable to the FeRh(30 nm)/Pt(3.5 nm) sample at low temperature. Similarly to the FeRh/Pt sample, we see that the residual magnetisation is never zero but starts increasing slightly below 200 K. Polarized neutron reflectivity measurements have shown that this residual magnetism mainly resides at the interface with the MgO substrate and the top interface. Residual magnetism at the bottom interface does not have a strong dependence on temperature [18]. Residual magnetism at the top surface, which is of interest for the interpretation of our THz emission measurements, can be caused by alloying and therefore depends on the capping material and unsually has a stronger dependence on temperature [14, 19, 20]. In our case, however, we don not observe a significant difference in the SQUID data of capped and uncapped FeRh and we therefore tend to associate the ferromagnetic top-layer with ferromagnetic FePd that forms independently on capping. Previous works have shown that for uncapped FeRh and FeRh capped with Pt, ferromagnetism at the top-surface is negligible [19, 21]. Differently from these works, our FeRh is doped with 2.8% Pd at the top interface.

Fig. S7 (b) shows the THz emission amplitude as a function of temperature. As discussed in the main text, in the FM phase the THz emission has a magneto-dipole nature due to the reduced contribution from the spin-Hall effect. In the AF phase we still observe a non-zero THz emission, odd with respect to sample flipping. We explain this with the non-negligible spin-Hall angle of AF-FeRh.

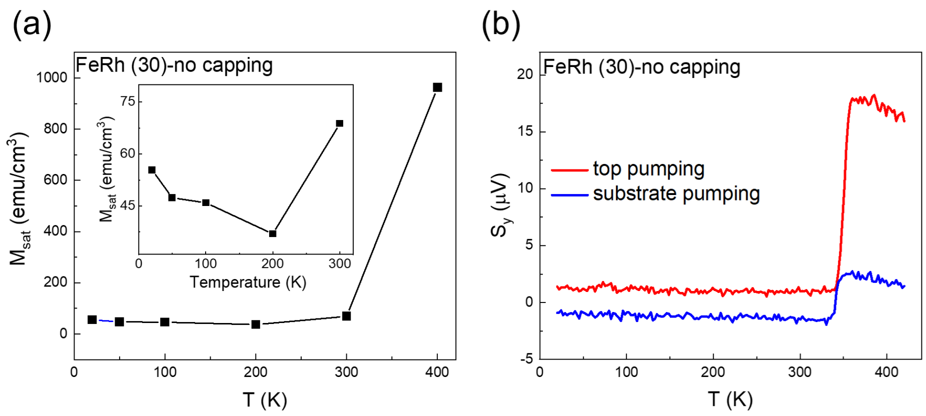

Fig. S 7 – Temperature dependence of the magnetisation and THz emission amplitude for the uncapped FeRh sample. **(a)** Temperature dependence of the saturation magnetisation M_{sat} extracted from SQUID magnetometry measurements for an uncapped FeRh(30 nm) film. The inset represents a zoom-in of the low temperature region below 300 K. **(b)** Temperature dependence of the THz emission amplitude in uncapped FeRh(30 nm). An optical fluence of 1.2 mJ/cm^2 was used and an in-plane field of 860 mT was applied during the measurement. The two curves represent measurements performed for the sample pumped from the substrate side (blue curve) and top surface (red curve).

F. Optical fluence dependence of THz emission from FeRh(30)/Pt(3.5)

Fig. S8 shows how the THz emission from FeRh(30)/Pt(3.5) depends on the optical pump fluence. Normalised temperature dependence data presented in (c) demonstrates that the low temperature THz emission enhancement is qualitatively identical for all studied fluences as all curves overlap in the 20-300 K range. In (b), we show that the magnitude of S_y^* is linear with the fluence at temperatures in the 20-300 K range, and that no threshold behaviour is observed. These results exclude that the THz enhancement at low temperatures is due to a phase transition induced by transient pump heating. In Fig. S6(d) we focus instead on the FM-AF transition region and show that as the pump fluenece is increased the hysteresis closes, in agreement with previous works [22].

G. Dependence of THz emission on FeRh doping in FeRh(30)/Pt(3.5) structures

According to our microscopic model, residual ferromagnetism at the interface with Pt plays a key role in triggering spin pumping from antiferromagnetic FeRh. This ferromagnetism will depend on the chemical composition and doping level of the interface [23]. We have therefore compared spin pumping in two different samples with the following composition:

FeRh35-Pt: MgO/$Fe_{50}Rh_{46.8}Pd_{1.7}Ir_{1.5}$(5)/$Fe_{50}Rh_{47.1}Pd_{2.2}Ir_{0.7}$(10)/$Fe_{50}Rh_{47.2}Pd_{2.8}$(15)/Pt(3.5)

FeRh36-Pt: MgO/$Fe_{50}Rh_{47.2}Pd_{2.8}$(15)/$Fe_{50}Rh_{47.1}Pd_{2.2}Ir_{0.7}$(10)/$Fe_{50}Rh_{46.8}Pd_{1.7}Ir_{1.5}$(5)/Pt(3.5)

The two samples have identical doping levels but the doping gradient is inverted. In this way we guarantee very similar bulk properties (crystal quality and conductivity, which determines the THz outcoupling), but different interface properties. In Fig. S9 we compare the temperature dependence of the THz emission amplitude S_y and spin pumping S_y^* in the two samples, normalised to the values in the ferromagnetic phase. First, we notice that in sample FeRh35-Pt, the phase transition is shifted to higher temperatures. This is in agreement with the fact that Ir-rich FeRh has a higher transition temperature than Pd-rich FeRh [24] and confirms that only the volume closer to the Pt interface is involved in the spin-pumping. Also, we see that as temperature is decreased below the transition temperature, we measure a lower value of the spin pumping in FeRh35-Pt. This shows that the chemical composition of the interface with Pt is important. The fact that spin pumping decreases with Pd concentration provides further evidence that residual magnetism resides in FePd alloying.

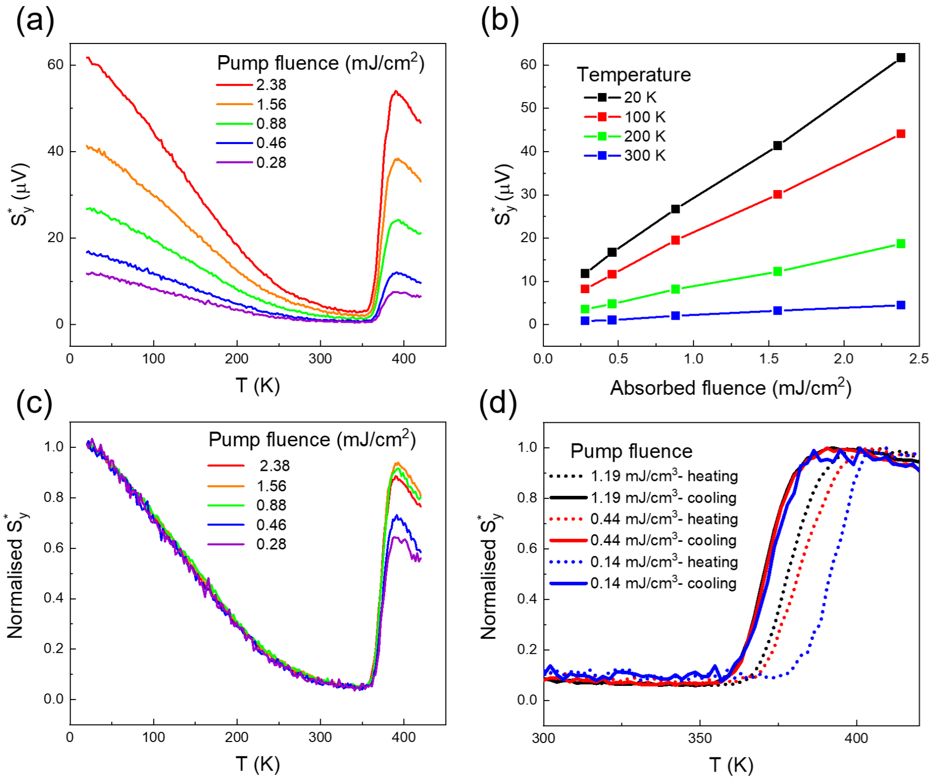

Fig. S 8 – Optical fluence dependence of THz emission from FeRh(30)/Pt(3.5). **(a)** Temperature dependence of S_y^* for different pump fluences between 0.28 and 2.38 mJ/cm^2. **(b)** S_y^* plotted as a function of pump fluence at selected temperatures between 20 and 300 K. **(c)** Data sets from **(a)** normalised with respect to the values of S_y^* at 20 K. **(d)** Temperature dependence of normalised S_y^* for different pump fluences. Both cooling and heating branches are shown.

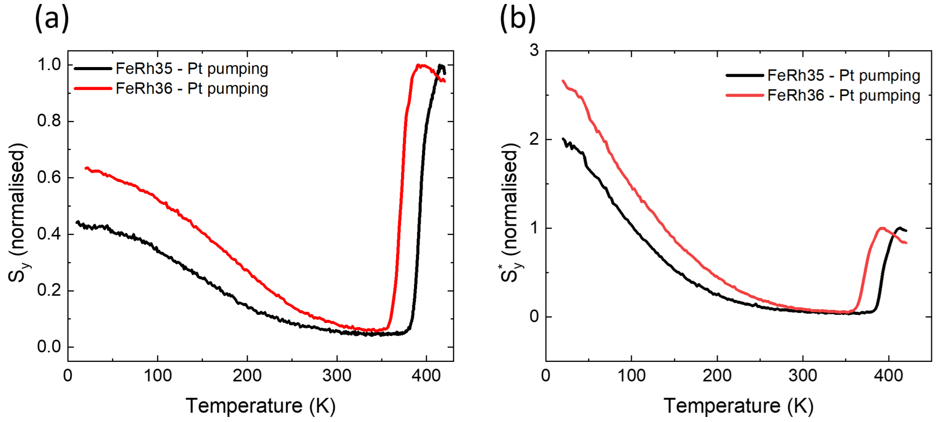

Fig. S 9 – Temperature dependence of S_y **(a)** and S_y^* **(b)** when pumping from the Pt side in sample FeRh35-Pt (black) and FeRh36-Pt (red). The pump fluence is 2.38 mJ/cm^2 and a magnetic field of 860 mT is applied.

H. THz transmission through FeRh(30)/Pt(3.5

Fig. S10 shows the THz transmissivity of the FeRh-Pt sample as a function of temperature. θ is defined as the ratio between the transmitted THz radiation and the reference signal measured with no sample in the propagation path at 1 THz. Changes in the structural and electronic properties of FeRh across the AF-FM transition result in a

steep variation of θ around $T_{(\mathrm{AF-FM})}$. The phase transition region displays a clear temperature hysteresis between the cooling and heating branches, as expected for a first-order phase transition. Below $T_{(\mathrm{AF-FM})}$, θ continuously decreases, which is attributed due to the increasing conductivities of FeRh and Pt [25, 26].

However, if we compare the value of $T_{(\mathrm{AF-FM})}$ measured via THz transmission and optical pump-THz emission (red dotted line) we observe that this is about 10 K lower in the second case, which could be explained by a small amount of accumulated heat when the sample is pumped optically.

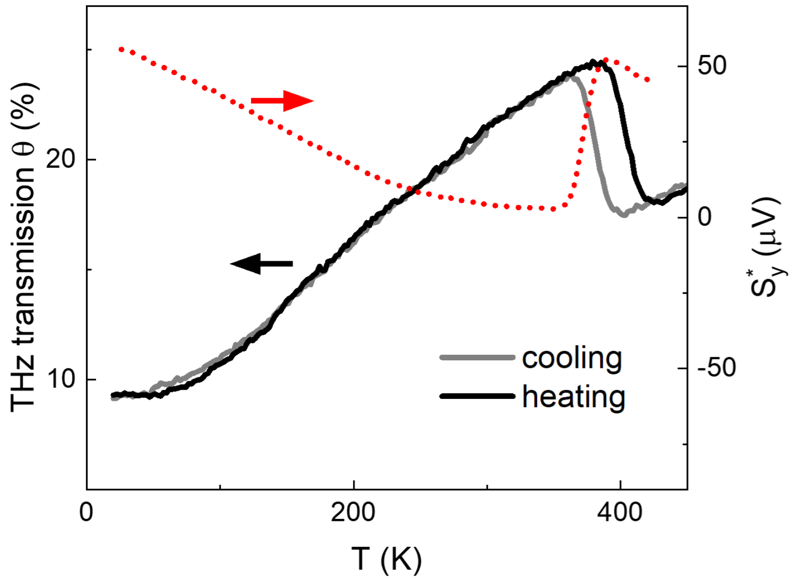

Fig. S 10 – Transmission of 1 THz field through FeRh(30)/Pt(3.5) for Pt incidence. Cooling (grey) and heating (black) branches are indicated. The experiment was performed with an external magnetic field of 860 mT continuously applied in-plane. The red dotted line indicates the temperature dependence of S_y^* when cooling from high to low temperature. The pump fluence is 2.38 mJ/cm2 and a magnetic field of 860 mT is applied.

I. Comparison with MgO/CoFeB(30)/Pt(5) emitter

Here we compare the temperature dependence of the THz emission in FeRh(30)/Pt(3.5) and CoFeB(30)/Pt(5). The magnetisation of CoFeB varies only slightly with temperature in the explored temperature range (Fig. S11 (a)), while the decrease in THz transmissivity as temperature is lowered reflects the increase in conductivity, similarly to the FeRh sample (Fig. S10). The THz emission (Fig. S 11 (c)-(d)), although comparable in magnitude, follows a very different temperature dependence in the two samples, decreasing with decreasing temperature in the CoFeB sample.

S4. GENERALISED ATOMISTIC SPIN DYNAMICS MODEL

In the four-spin FeRh model in S2 B the Rh moments are not explicitly represented, only the Fe moments are simulated. This means we cannot simulate the effect of Rh moments being induced by the laser pulse. We therefore develop a second spin model within the JAMS software package, where the dynamics of both the Fe and Rh moments are solved.

The FeRh crystal has the space group Pm$\bar{3}$m (221). We perform calculations using the cubic cell with the basis vectors

$$\begin{aligned}\mathbf{a} &= a_0(1,0,0),\\ \mathbf{b} &= a_0(0,1,0),\\ \mathbf{c} &= a_0(0,0,1),\end{aligned} \tag{6}$$

with the lattice constant $a_0 = 3.0$ Å. In the cubic cell, there is one Fe atom and one Rh atom. These are located at

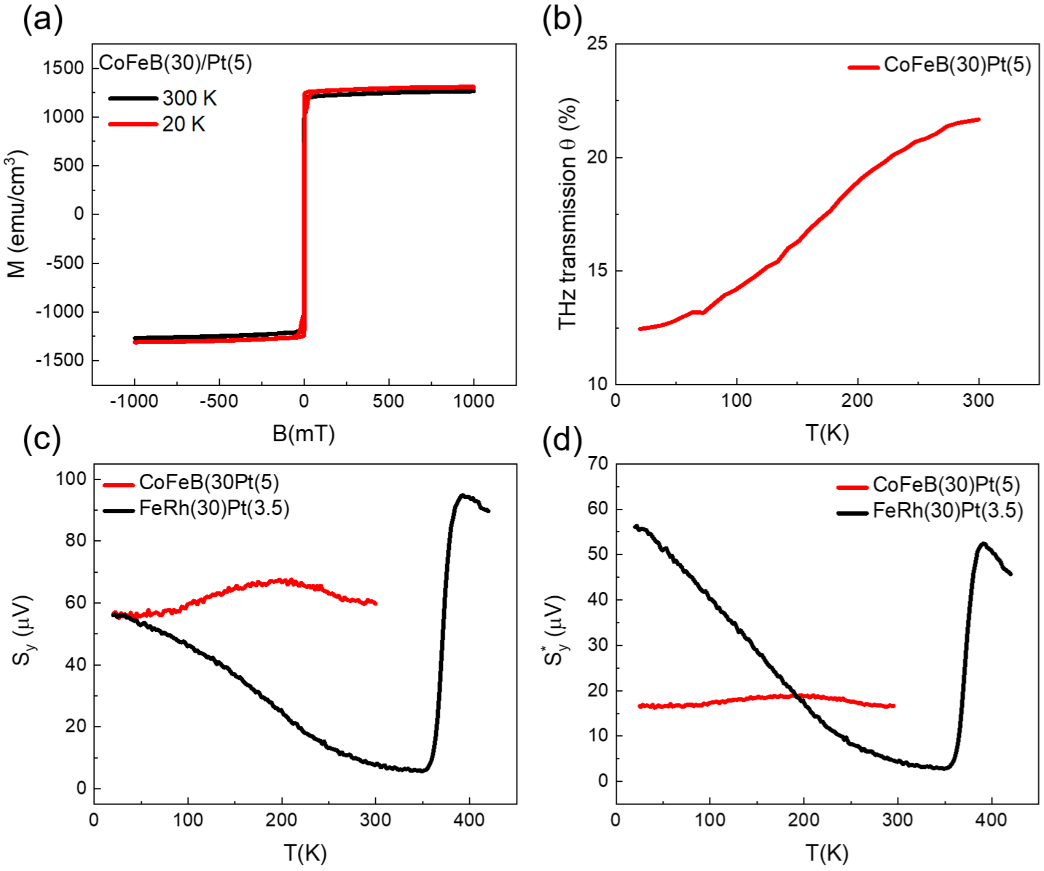

Fig. S 11 – Comparison between FeRh and CoFeB. **(a)** Magnetic hysteresis loops of CoFeB(30)/Pt(5) measured by SQUID at 300 K (black) and 20 K (red). **(b)** Transmission of 1 THz field through CoFeB(30)/Pt(5) for Pt incidence. The experiment was performed with an external magnetic field of 860 mT continuously applied in-plane. **(c)** Temperature dependence of the THz emission amplitude S^y in CoFeB(30)/Pt(5) (red) and FeRh(30/Pt(3.5) (black). The measurement was done applying a 860 mT magnetic field and pumping the sample from the Pt side and measuring the THz emission behind the substrate. **(d)** Temperature dependence of the THz emission amplitude rescaled by the optical absorption and THz outcoupling, S_y^*, in top-pumping geometry with an applied magnetic field of 860 mT for CoFeB(30)/Pt(5) (red) and FeRh(30)/Pt(3.5) (black).

(in fractional coordinates)

$$\begin{aligned} \mathrm{Fe} &= (0.0, 0.0, 0.0), \\ \mathrm{Rh} &= (0.5, 0.5, 0.5). \end{aligned} \tag{7}$$

The Hamiltonian is

$$\mathcal{H} = -\frac{1}{2}\sum_{ij} J_{ij}\mathbf{S}_i \cdot \mathbf{S}_j - \frac{1}{2}\sum_{ij\in Fe} B_{ij}(\mathbf{S}_i \cdot \mathbf{S}_j)^2 - \sum_{i\in \mathrm{Fe}} k(\hat{\mathbf{e}} \cdot \mathbf{S}_i)^2 + \sum_i L_{2,i}|S_i|^2 + L_{4,i}|S_i|^4 + L_{6,i}|S_i|^6, \tag{8}$$

where $\vec{S}_i$ are dimensionless classical vectors which can change in length. The first term is the Heisenberg exchange, where J_{ij} are the interaction energies. We used values from Polesya et al. [27] for Fe-Fe interactions and Fe-Rh interactions. Rh-Rh interactions are considered to be negligible. The second term in the Hamiltonian is a biquadratic exchange between Fe sites, which is necessary for the metamagnetic phase transition [27, 28]. The factors of 1/2 account for the double counting of each bond. The third term is the uniaxial anisotropy energy of the Fe sites with an axis $\hat{\mathbf{e}}$. The final term is a Landau Hamiltonian that describes the energetics of changes in the length of spin moments, where L_2, L_4, L_6 are parameters with units of energy. In our model of FeRh the Fe moments have a stable minimum at unit length. The Rh moments are unstable and contain only an L_2 term. This means that the minimum energy for the Rh Landau term is for zero spin length. However, the ferromagnetic Fe-Rh interactions compete with

this and cause a Rh moment to form if there is a net Fe magnetisation. The values we used in our model Hamiltonian are given in Supplementary Table 4.

We use the generalised Langevin spin equation [29]

$$\frac{\partial \mathbf{S}_i}{\partial t} = -\gamma \mathbf{S}_i \times \mathbf{B}_i + \gamma\lambda \mathbf{B}_i + \boldsymbol{\xi}_i \tag{9}$$

where $\mathbf{B}_i = -(1/\mu_i)\nabla_{\mathbf{S}_i}\mathcal{H}$ is the effective field in teslas with μ_i the magnetic moment on site i in Bohr magnetons, μ_{B}. ξ_i describes stochastic processes of the Langevin thermostat. λ is a dimensionless damping parameter which describes the coupling to the bath. Here we use a quantum thermostat, the correlations of ξ_i obey

$$\langle \xi_{i,a}(t)\rangle = 0; \quad \langle \xi_{i,a}(t)\xi_{j,b}(t')\rangle_\omega = \delta_{ij}\delta_{ab}\frac{2\lambda k_{\mathrm{B}}T}{\gamma\mu_i}\frac{\hbar\omega}{\exp\left(\hbar\omega/k_{\mathrm{B}}T\right)-1}, \tag{10}$$

where a, b are cartesian components (x, y, z), k_{B} is Boltzmann's constant, T is the temperature in kelvins. $\langle\cdots\rangle_\omega$ indicates the correlation function is defined in frequency (ω) space. Further details of the quantum thermostat implementation can be found in Ref. 11. We integrate (9) numerically using the Runge-Kutta fourth order method with a timestep of $\Delta t = 0.1$ fs. The values of the material parameters used for FeRh are given in Supplementary Table 3.

To simulate the effect of a transient spin current inducing a Rh moment, we increase the size of the Rh moment at the beginning of each time step using a temporal Gaussian pulse

$$\mathbf{S}'_{i,\mathrm{Rh}} = \mathbf{S}_{i,\mathrm{Rh}} + \frac{\Delta}{2\pi\sigma}\exp\left(-\frac{(t-t_0)^2}{2\sigma^2}\right)\hat{\mathbf{J}}, \tag{11}$$

which has a width of σ in dimensions of time, a temporal center of t_0, a dimensionless amplitude of Δ and $\hat{\mathbf{J}}$ is a unit vector in the direction of the polarisation of the incoming spin current.

Within the model we assume that the FeRh Néel vector is at 45 degrees to the [100] (along x in Fig. 1(a) of the main text) direction [30, 31] along which the field is applied and the ferromagnetic regions will align. Therefore, $\hat{\mathbf{e}} = (\sqrt{2}/2, \sqrt{2}/2, 0)$ and the transient spin current is polarised along $\hat{\mathbf{J}} = (1, 0, 0)$.

Supplementary Table 3 – Model parameters for the generalised Langevin spin equation (9).

symbol	value	units
γ	0.17608596	$\mathrm{rad}\cdot\mathrm{ps}^{-1}\cdot\mathrm{T}^{-1}$
λ	0.1	(dimensionless)
μ_{Fe}	3.2[d]	μ_{B}
μ_{Rh}	1.0[d]	μ_{B}

[d] Ref. 27

S5. ESTIMATE OF RH EXCHANGE FIELD

The sudden appearance of the Rh moment couples to the Fe through a strong exchange interaction. For the Fe in the AF state to generate a net moment the system must overcome the critical field for a spin-flop transition. The exchange field exists for such a short time that the spin-flop state is only transient, but a net magnetisation is produced (Fig.4b main text – there is a net Fe magnetisation) in the AF and a corresponding spin pumping. We can put this on a more quantitative footing. The FeRh AFM state is dominated by the antiferromagnetic J_5 interaction, and the effective exchange field felt by the Fe sublattices has a field strength,

$$B_E = \frac{8|J_5|}{\mu_{\mathrm{Fe}}}. \tag{12}$$

The anisotropy field strength is,

$$B_A = \frac{2k}{\mu_{\mathrm{Fe}}}. \tag{13}$$

Supplementary Table 4 – Exchange constants for the Hamiltonian (8). A single interaction vector is given in fractional coordinates; equivalent vectors can be generated from the m$\bar{3}$m point group symmetry operations. The interaction distances are given in units of the lattice constant (a).

symbol	types	vector (fractional coordinates)	number	distance (lattice constants a_0)	value (meV)
J_1	Fe-Rh	$(1/2, 1/2, 1/2)$	8	0.866025	27.16[a]
J_2	Fe-Fe	(0, 0, 1)	6	1.000000	−3.90[b]
J_3	Fe-Fe	(0, 1, 1)	12	1.414214	5.84[b]
J_4	Fe-Rh	$(1/2, 1/2, 3/2)$	24	1.658312	1.54[a]
J_5	Fe-Fe	(1, 1, 1)	8	1.732051	−16.34[b]
J_6	Fe-Fe	(0, 0, 2)	6	2.000000	2.76[b]
J_7	Fe-Fe	(0, 1, 2)	24	2.236068	0.80[b]
J_8	Fe-Fe	(1, 1, 2)	24	2.449490	−1.32[b]
B_1	Fe-Fe	(0, 0, 1)	6	1.000000	5.00
k	Fe	-	-	-	0.06242
L_2	Fe	-	-	-	−440.987[c]
L_4	Fe	-	-	-	150.546[c]
L_6	Fe	-	-	-	50.6794[c]
L_2	Rh	-	-	-	100.64
L_4	Rh	-	-	-	0.0
L_6	Rh	-	-	-	0.0

[a] Ref. 27, Fig. 4b
[b] Ref. 27, Fig. 4a, 'DLM' dataset
[c] Ref. 32

The lower bound for the critical field of a two sublattice antiferromagnet with easy axis (at $T = 0$) is [33],

$$B_0 > \sqrt{B_A(2B_E - B_A)} \tag{14}$$

where B_0 is a field strength applied along the easy axis. For our model of FeRh $B_0 \approx 30$ Tesla. Our applied field is much lower than this, meaning that the two AFM modes can be split only a small amount, leading to only a small spin current, proportional to the applied field as seen in normal AFs. However, the spontaneously generated Rh moment causes the sudden appearance of an additional exchange field inside of the AFM. This field is not staggered (alternating between sublattices) but directional, due to the spin polarised current. Hence, it acts on the antiferromagnetic Fe sublattices in a similar way to an applied field, but is much stronger. The field strength is approximately,

$$B_{\mathrm{Rh}} = \frac{8|J_1|}{\mu_{\mathrm{Fe}}} S_{\mathrm{Rh}}. \tag{15}$$

Our results show that we only generate $S_{\mathrm{Rh}} = 0.2\mu_{\mathrm{B}}/1.0\mu_{\mathrm{B}}$ (Fig. 4b main text), but nevertheless the peak Fe-Rh exchange field is therefore $B_{\mathrm{Rh}} = 235$ Tesla, much greater than the spin-flop field. This explains why the Fe develops a net magnetisation, it is trying to spin-flop, although does not have enough time to fully transition in the 1 ps the field appears for. This also explains why the spin pumping is far larger than can be achieved from an applied field below the spin-flop field of an antiferromagnet, and more similar to the large change seen in spin pumping once the field is above the spin flop transition [34].

[1] S. J. Byrnes, Multilayer optical calculations, arXiv:1603.02720.
[2] R. Stephens and I. Malitson, Index of refraction of magnesium oxide, J. Res. Natl. Bureau Stand. **49**, 249 (1952).
[3] W. S. M. Werner, K. Glantschnig, and C. Ambrosch-Draxl, Optical Constants and Inelastic Electron-Scattering Data for 17 Elemental Metals, J. Phys. Chem. Ref. Data **38**, 1013 (2009).
[4] V. Saidl et al., Investigation of magneto-structural phase transition in FeRh by reflectivity and transmittance measurements in visible and near-infrared spectral region, New J. Phys. **18**, 083017 (2016).
[5] Courtesy of Karel Vyborny.
[6] T. Seifert et al., Efficient metallic spintronic emitters of ultrabroadband terahertz radiation, Nat. Photonics **10**, 483 (2016).
[7] D. M. Nenno et al., Modification of spintronic terahertz emitter performance through defect engineering, Sci. Rep. **9**, 13348 (2019).
[8] M. A. de Vries, M. Loving, A. P. Mihai, L. H. Lewis, D. Heiman, and C. H. Marrows, Hall-effect characterization of the metamagnetic transition in FeRh, New J. Phys. **15**, 013008 (2013).
[9] Y. Wang, P. Deorani, X. Qiu, J. H. Kwon, and H. Yang, Determination of intrinsic spin Hall angle in Pt, Appl. Phys. Lett. **105**, 152412 (2014).
[10] R. F. L. Evans, W. J. Fan, P. Chureemart, T. A. Ostler, M. O. A. Ellis, and R. W. Chantrell, Atomistic spin model simulations of magnetic nanomaterials, J. Phys.: Condens. Matter **26**, 103202 (2014); Vampire v6.0 (2023).
[11] J. Barker and R. W. Chantrell, Higher-order exchange interactions leading to metamagnetism in FeRh, Phys. Rev. B **92**, 094402 (2015).
[12] T. A. Ostler, C. Barton, T. Thomson, and G. Hrkac, Modeling the thickness dependence of the magnetic phase transition temperature in thin FeRh films, Phys. Rev. B **95**, 064415 (2017).
[13] J. R. Massey et al., Phase boundary exchange coupling in the mixed magnetic phase regime of a Pd-doped FeRh epilayer, Phys. Rev. Mater. **4**, 024403 (2020).
[14] R. Fan et al., Ferromagnetism at the interfaces of antiferromagnetic FeRh epilayers, Phys. Rev. B **82**, 184418 (2010).
[15] F. de la Peña et al., HyperSpy 10.5281/zenodo.4294676 (2020).
[16] S. A. Nepijko and G. Schönhense, Quantitative Lorentz transmission electron microscopy of structured thin permalloy films, Appl. Phys. A **96**, 671 (2009).
[17] P. Champness, G. Cliff, and G. Lorimer, Quantitative analytical electron microscopy of metals and minerals, Ultramicroscopy **8**, 121 (1982).
[18] T. P. Almeida et al., Direct visualization of the magnetostructural phase transition in nanoscale FeRh thin films using differential phase contrast imaging, Phys. Rev. Mater. **4**, 034410 (2020).
[19] C. Baldasseroni et al., Temperature-driven nucleation of ferromagnetic domains in FeRh thin films, Appl. Phys. Lett. **100**, 262401 (2012).
[20] Y. Ding et al., Bulk and near-surface magnetic properties of FeRh thin films, J. Appl. Phys. **103**, 07B515 (2008).
[21] C. Baldasseroni et al., Effect of capping material on interfacial ferromagnetism in FeRh thin films, J. Appl. Phys. **115**, 043919 (2014).
[22] N. Awari et al., Monitoring laser-induced magnetization in FeRh by transient terahertz emission spectroscopy, Appl. Phys. Lett. **117**, 122407 (2020).
[23] M. Jiang, X. Chen, X. Zhou, Y. Wang, F. Pan, and C. Song, Influence of film composition on the transition temperature of FeRh films, J. Cryst. Growth **438**, 19 (2016).
[24] C. Le Graët et al., Temperature controlled motion of an antiferromagnet- ferromagnet interface within a dopant-graded FeRh epilayer, APL Mater. **3**, 10.1063/1.4907282 (2015).
[25] R. E. Glover and M. Tinkham, Conductivity of Superconducting Films for Photon Energies between 0.3 and $40kT_c$, Phys. Rev. **108**, 243 (1957).
[26] T. J. Huisman, R. V. Mikhaylovskiy, A. Tsukamoto, T. Rasing, and A. V. Kimel, Simultaneous measurements of terahertz emission and magneto-optical Kerr effect for resolving ultrafast laser-induced demagnetization dynamics, Phys. Rev. B **92**, 104419 (2015).
[27] S. Polesya, S. Mankovsky, D. Ködderitzsch, J. Minár, and H. Ebert, Finite-temperature magnetism of FeRh compounds, Phys. Rev. B **93**, 024423 (2016).
[28] P. M. Derlet, Landau-Heisenberg Hamiltonian model for FeRh, Phys. Rev. B **85**, 174431 (2012).
[29] P.-W. Ma and S. L. Dudarev, Longitudinal magnetic fluctuations in Langevin spin dynamics, Phys. Rev. B **86**, 054416 (2012).
[30] Y. Xie et al., Effect of epitaxial strain and lattice mismatch on magnetic and transport behaviors in metamagnetic FeRh thin films, AIP Adv. **7**, 056314 (2017).
[31] Y. Xie et al., Magnetocrystalline anisotropy imprinting of an antiferromagnet on an amorphous ferromagnet in FeRh CoFeB heterostructures, NPG Asia Mater. **12**, 67 (2020).
[32] M. O. A. Ellis, M. Galante, and S. Sanvito, Role of longitudinal fluctuations in $L1_0$FePt, Phys. Rev. B **100**, 214434 (2019).
[33] A. G. Gurevich and G. A. Melkov, *Magnetization Oscillations and Waves* (CRC Press, 1996).
[34] S. M. Wu et al., Antiferromagnetic Spin Seebeck Effect, Phys. Rev. Lett. **116**, 097204 (2016).
