# Supplementary material for: Ultra-high spin emission from antiferromagnetic FeRh
Source: Nat Commun. 2024 Jun 11;15:4958. doi: 10.1038/s41467-024-48795-z (PMC11166917; doi:10.1038/s41467-024-48795-z)
Supplement: Supplementary file 3 — Reporting Summary [file 41467_2024_48795_MOESM3_ESM.pdf]

natureresearch

Corresponding author(s): Chiara Ciccarelli

Lasing Reporting Summary

Nature Research wishes to improve the reproducibility of the work that we publish. This form is intended for publication with all accepted papers reporting claims of lasing and provides structure for consistency and transparency in reporting. Some list items might not apply to an individual manuscript, but all fields must be completed for clarity.

For further information on Nature Research policies, including our data availability policy, see Authors & Referees.

• Experimental design

Please check: are the following details reported in the manuscript?

1. Threshold

Item	Yes/No	Details
Plots of device output power versus pump power over a wide range of values indicating a clear threshold	[x] Yes [ ] No	Fig.S8(b) of the supplementary information - no threshold behavior observed

2. Linewidth narrowing

Item	Yes/No	Details
Plots of spectral power density for the emission at pump powers below, around, and above the lasing threshold, indicating a clear linewidth narrowing at threshold	[ ] Yes [x] No	spectral power determined by set-up, does not provide useful information on the probed mechanism.
Resolution of the spectrometer used to make spectral measurements	[ ] Yes [x] No	NA

3. Coherent emission

Item	Yes/No	Details
Measurements of the coherence and/or polarization of the emission	[x] Yes [ ] No	Fig.1(c) of main text

4. Beam spatial profile

Item	Yes/No	Details
Image and/or measurement of the spatial shape and profile of the emission, showing a well-defined beam above threshold	[ ] Yes [x] No	does not provide useful information on the probed mechanism. Samples are homogeneous within the probed area.

5. Operating conditions

Item	Yes/No	Details
Description of the laser and pumping conditions *Continuous-wave, pulsed, temperature of operation*	[x] Yes [ ] No	In the section "Optical Pump-THz emission from FeRh-Pt" of the main article and the Methods.
Threshold values provided as density values (e.g. W cm^{-2} or J cm^{-2}) taking into account the area of the device	[x] Yes [ ] No	n the section "Optical Pump-THz emission from FeRh-Pt" of the main article.

6. Alternative explanations

Item	Yes/No	Details
Reasoning as to why alternative explanations have been ruled out as responsible for the emission characteristics *e.g. amplified spontaneous, directional scattering; modification of fluorescence spectrum by the cavity*	[x] Yes [ ] No	In "Discussion" of main article and supplementary information

7. Theoretical analysis

Item	Yes/No	Details
Theoretical analysis that ensures that the experimental values measured are realistic and reasonable *e.g. laser threshold, linewidth, cavity gain-loss, efficiency*	[x] Yes [ ] No	In "Discussion" of the main article and sections S4 and S5 of the Supplementary Information.

8. Statistics

Item	Yes/No	Details
Number of devices fabricated and tested	[x] Yes [ ] No	In the section "Optical Pump-THz emission from FeRh-Pt" of the main article and the Methods.
Statistical analysis of the device performance and lifetime (time to failure)	[ ] Yes [x] No	Not applicable for these devices

March 2018
